# Attenuation of Chilling Injury and Improving Antioxidant Capacity of Persimmon Fruit by Arginine Application

**DOI:** 10.3390/foods11162419

**Published:** 2022-08-11

**Authors:** Fahimeh Nasr, Farhang Razavi, Vali Rabiei, Gholamreza Gohari, Sajid Ali, Christophe Hano

**Affiliations:** 1 Department of Horticulture, Faculty of Agriculture, University of Zanjan, Zanjan 45371-38791, Iran; 2 Department of Horticulture, Faculty of Agriculture, University of Maragheh, Maragheh 83111-55181, Iran; 3 Department of Horticulture, Faculty of Agricultural Sciences and Technology, Bahauddin Zakariya University, Multan 60800, Pakistan; 4 Laboratoire de Biologie des Ligneux et des Grandes Cultures (LBLGC), INRAE USC1328, Université d’Orléans, 28000 Chartres, France

**Keywords:** persimmon, natural compounds, L-arginine, total carotenoid, soluble tannin

## Abstract

Persimmon is a climacteric perishable fruit with a short storage life. In recent years, using natural compounds that are safe for human health and environment have obtained more attention in postharvest investigations. The current research was conducted to study efficacy of postharvest L-arginine treatment at 0, 0.3, and 0.6 mM in improving chilling tolerance and maintaining the nutritional quality of persimmon fruit during low-temperature storage. According to the results, the highest weight loss (4.3%), malondialdehyde (MDA (5.8 nmol g^−1^ FW)), and hydrogen peroxide (H_2_O_2_ (22.33 nmol g^−1^ FW)) was detected in control fruit. Fruit firmness was gradually decreased during storage, but it was slower in L-arginine-treated fruit. The highest tissue firmness (3.8 kg cm^−2^) was noted in fruit treated with 0.6 mM L-arginine. The chilling was gradually increased during storage. Fruits treated with L-arginine showed a lower chilling injury than the control fruit. Total soluble tannin compound and antioxidant enzymes activities in persimmons declined during cold storage. L-arginine treatment significantly maintained antioxidant enzymes activity, antioxidant capacity, and total soluble tannin compounds, while L-arginine had no significant impact on titratable acidity and total soluble solids. It seems that a reduction in oxidative damage and an increase in quality of persimmon during low-temperature storage manifested several defense mechanisms induced by exogenous application of L-arginine. These findings indicated that the application of L-arginine to maintain the quality and increase postharvest life of persimmon is very useful and can be applied during cold storage.

## 1. Introduction

Persimmon (*Diospyrus kaki* L.) is an imperative fruit from the Ebenaceae family. The fruit is preferred because of its delicious taste, attractive color, nutritive value, and bioactive compounds such as phenolic compounds and flavonoids, which act as antioxidants. One of the main reasons for postharvest loss of persimmon fruits is because of susceptibility to chilling injury (CI) when stored at cold storage. Below 4 °C, persimmon fruits show symptoms such as loss firmness, peel browning, discoloration, and decay [1]. A common method to maintain quality and prevent chilling injury of postharvest persimmon is using different treatments such as carboxymethyl-chitosan [2], heat shock [3], hydrogen sulfide [4], methyl jasmonate [5], 1-methylcyclopropene [6], and pulsed-light treatments [7] during cold storage. Due to the unsafe effects of the chemicals on the environment and human health, the use of these materials has been recently limited, and it is crucial to use the harmless compounds such as arginine in the postharvest technology of horticultural crops [8,9].

Postharvest researches have revealed that the application of arginine can extend the storage life of horticultural crops by delaying the ripening or senescence process. L-arginine is an α-amino acid that is applied in the biosynthesis of proteins. L-arginine is a precursor in the biosynthesis of polyamines and signaling molecules such as nitric oxide [10]. Nitric oxide is the main signaling molecule involved in the physiological processes of the plant [11]. It has also been indicated that arginine protects plant cells against oxidative stress by biosynthesis nitric oxide and reducing ROS accumulation [12]. Arginine could increase tolerance against chilling and reduce occurrence of chilling injury in different fruits and vegetables [8,13]. L-arginine plays a consequential role in the activity of different enzymes in fruits. This amino acid is attached to membrane phospholipids and nucleic acid and improves the activity of enzymes such as catalase. The exogenous application of arginine in pistachio [9], tomato [14], strawberry [10], and pomegranate fruit [8] also reduced postharvest decay and inhibited postharvest senescence. In fresh-cut apple and lettuce, L-arginine treatment improved stress resistance, inhibited browning, and did not affect the taste of fruit during the postharvest period [15]. In another study, the use of L-arginine at 0.05 mmol L⁻¹ concentration reduced the decay, MDA, and weight loss and increased antioxidant capacity in green asparagus during cold storage [16]. Li et al. [13] reported that application of L-arginine 10 mM increased phenol and flavonoid accumulation; maintained firmness; and delayed increase of electrolyte leakage, browning, and PPO activities in mushrooms compared to the control. It has been illustrated that L-arginine 200 μmol/L treatments significantly decreased fruit decay as well as MDA accumulation and effectively increased peroxidase, β-1,3-glucanase, phenylalamine ammonialyase, chitinase activities and lignin, phenols, and flavonoid content in winter jujube fruits during storage [17]. Furthermore, arginine treatment significantly decreased electrolyte leakage and weight loss and increased flavor, firmness, ascorbic acid, and tolerance in chilling injury in cucumber stored in 5 °C [12]. According to the previous research, L-arginine has a great effect on alleviating chilling injury and preserving nutritional quality of fruits at low-temperature storage. However, the effect of L-arginine on persimmons is a novel field that has not been researched yet, so this study aimed to explore the potential effect of L-arginine application on improving fruit quality, mitigating chilling injury, and increasing the storage life of persimmon fruit under cold stress.

## 2. Materials and Methods

### 2.1. Plant Material and Treatment of L-arginine

In order to explore the effect of L-arginine in increasing storage life and postharvest quality of persimmon fruit, an experiment was carried out in the post-harvest lab and refrigerator at the University of Zanjan. Persimmon (*Diospyrus kaki* Thunb.) fruits (210 fruit) were picked from a commercial orchard in Karaj, Iran, and transferred to the postharvest laboratory. This study was performed as a factorial experiment based on a complete randomized design with three replicates per treatment. The first factor was L-arginine at three levels (0 (control), 0.3, and 0.6 mM), and the second factor was storage time at three levels of 15, 30, and 45 days. On the harvest day, 21 fruits were selected and analyzed for initial physicochemical attributes. Other samples (189 fruits) were randomly divided into 21 fruits for each treatment in three replications (7 fruits per replication) in each sampling date. Persimmon fruits were treated with 0 (control), 0.3, and 0.6 mM L-arginine for 10 min and then stored at 4 ± 1 °C and relative humidity 85–90% for 45 days. At 15 day intervals, one group was selected randomly and kept for shelf life of two days at 25 °C and various biochemical analyses were performed. 

### 2.2. Assessment of Browning Index

The percentages of the browning index were calculated by Wang et al. [18]. Tissue gelling and skin and tissue browning are the main signs of chilling injury in persimmon. In this study, the browning index was assessed by peel browning (PB) on each fruit by divided into 5 classes: 0 (no PB, excellent quality), 1 (<25% PB), 2 (25–50% PB), 3 (50–75% PB), and 4 (>75% PB).

The browning index = ∑ (PB level) × (Number of fruits at each PB level)/(4 × total number of fruit in the treatment)

### 2.3. Assessment of Malondialdehyde (MDA) and H_2_O_2_

MDA accumulation was analyzed according to the Heath and Packer [19] method. One g of fruit frozen tissue was ground in 15 mL 0.1% (*w*/*v*) trichloroacetic acid (TCA). Thereafter, centrifugation was performed at 10,000× *g* for 20 min. Next, supernatant 2 mL was reacted with 2 mL 0.1% TCA comprising 0.5% (*w*/*v*) thiobarbituric acid (TBA). The mixtures were then heated to 96 °C for 30 min, quickly cooled, and centrifugation was done at 10,000× *g* for 5 min. The supernatant was noted at 450, 532, and 600 nm, and MDA accumulation was computed using the given equation:

MDA = (6.45 × (A532 − A600) − 0.56 × A450) × V/W

where A600: absorbance at 600 nm; A532: absorbance at 532 nm; A450: absorbance at 450 nm; V: volume of extraction; and W: fresh weight of sample. 

The amount of H_2_O_2_ was assessed according to the Alexieva et al. [20] method, in which 0.5 g of frozen persimmon fruit samples were macerated in 5 mL ice-cold 3% trichloroacetic acid, and then, it was centrifuged at 12,000× *g* for 20 min at 4 °C, and the supernatants were used for H_2_O_2_ measurements. Then, 0.25 mL supernatants were mixed with 0.25 mL 100 mM phosphate buffer (pH 7.0) and 1 mL 1 M potassium iodide. The optical density of the H_2_O_2_ solution was recorded at 390 nm and reported as nmol g^−1^ FW.

### 2.4. Assessment of Firmness and Weight Loss

A handle OSK 1618 penetrometer equipped with an 8 mm tip was utilized for firmness measurement and reported kg cm^−2^. The weight loss value of fruit was determined by the digital scale model CANDGL300 and reported (%) [21].

### 2.5. Assessment of Titratable Acidity and Total Soluble Solids

A manual refractometer was used to measure the total soluble solids, and the sodium hydroxide titration method was used to measure the titratable acidity [22].

### 2.6. Assessment of Total Soluble Tannin

The amount of total soluble tannins is determined by using Folin–Denis assay. To do so, 1 g tissue was macerated in 80% methanol by means of a mortar. The amount was expressed as g kg^−1^ tannic acid and calculated from the prepared standard curve [23].

### 2.7. Assessment of Total Carotenoid

The total carotenoids content was assayed using Wang et al. [24] method. Fruit tissues (1 g) were macerated in 5 mL acetone–hexane 40/60 mixture. The layer of the upper phase was shifted into chilled test tubes, and the residual aqueous layer was re-extracted with aforementioned solution of acetone–hexane. The absorbance of all extracts was recorded at 450 nm, and its concentration was expressed as mg Kg^−1^ FW.

### 2.8. Assessment of Antioxidative Enzymes Activity

For analyzing catalase (CAT), superoxide dismutase (SOD), and ascorbate peroxidase (APX) enzymes activity, 5 g of frozen persimmon fruit tissues were ground in 50 mM phosphate buffer (pH 7.8) having 0.2 mM EDTA and 2% PVPP. Then, it was centrifuged at 12,000× *g* for 15 min at 4 °C, and supernatant was utilized for CAT, SOD, and APX activities measurements. CAT activity was assessed with Zhang et al. [25] procedure. The absorbance of the solution was measured at 240 nm and reported as U g^−1^ FW. SOD activity was assessed according to Zhang et al. [25]. The absorbance of the solution was recorded at 560 nm and reported as U g^−1^ FW. APX activity was assessed according to Zhang et al. [25]. The absorbances of the solutions were recorded at 290 nm and reported as U g^−1^ FW.

### 2.9. Assessment of Total Antioxidant Capacity

The 2,2′-diphenyl-1-picrylhydrazyl (DPPH) reagent method was utilized for total antioxidant capacity assessment. In brief, 1 g of frozen persimmons fruit tissues was ground in 8 mL of 0.1% HCl along with 80% methanol. Then, its centrifugation was performed at 10,000× *g* for 15 min, and 50 μL of the supernatants was reacted in 1.95 mL of DPPH (0.1 mM) made in the methanolic solution. Then, all mixtures were placed under darkness for 30 min under the conditions of ambient temperature. The absorbance of solutions was recorded at 517 nm and reported as % [26]. As a control, optical density of the blank solutions of DPPH was observed at 517 nm.

### 2.10. Statistical Analysis

Results were analyzed statistically with SPSS statistics version 22.0 (SPSS Inc., Chicago, IL, USA) software, and differences among means were measured by Duncan’s multiple range test for significance at *p* < 0.05.

## 3. Results and Discussion

### 3.1. Browning Index

**Figure 1:**
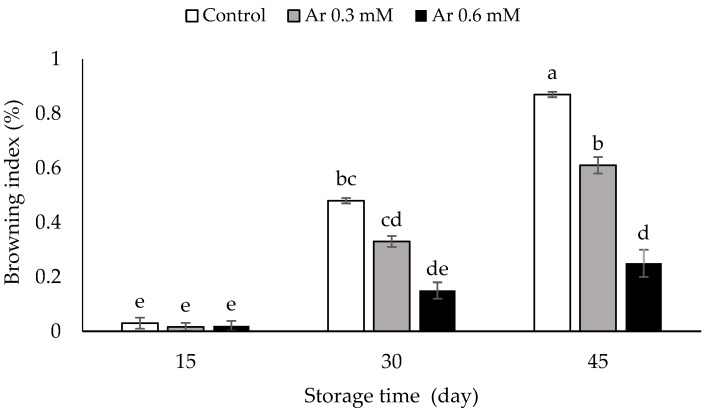
The effect of different levels of postharvest treatment of L-arginine on browning index of persimmon cv. “Karaj” fruits during cold storage (DMRT, *p* ≤ 0.05). The values of data are average of *n* = 3, and bar represents standard error of the means. Different letters above the bars show significant difference at *p* < 0.05 by Duncan’s multiple range test.

Based on the results, the browning rate was increased in the persimmons under storage. The treatment of L-arginine at the rates of 0.3 and 0.6 mM effectively prevented the browning index of the fruits versus the control. The lowest rate of browning was observed in the treatment of 0.6 mM L-arginine at 30 and 45 days after storage (DAS) (Figure 1).

Firmness loss and fruit internal and external browning are the chief symptoms of persimmon chilling damage. The persimmon fruit tissue is converted from a flexible crystalline liquid into a jelly structure by phase transition due to chilling damage [27,28]. Fruit browning during chilling happens by PPO and POD enzymes, which involves oxidation of phenolic substrates, and gelation during chilling happens by the decomposition of pectin polymers, and the tissue becomes much stickier than normally matured fruits [29]. L-arginine improves plant resistance to postharvest oxidative stress by synthesizing nitric oxide. Nitric oxide plays a role in plant resistance by increasing antioxidant enzymes activities and controlling free radicals, by which it reduces the browning of the fruit [30].

### 3.2. MDA and H_2_O_2_

**Figure 2:**
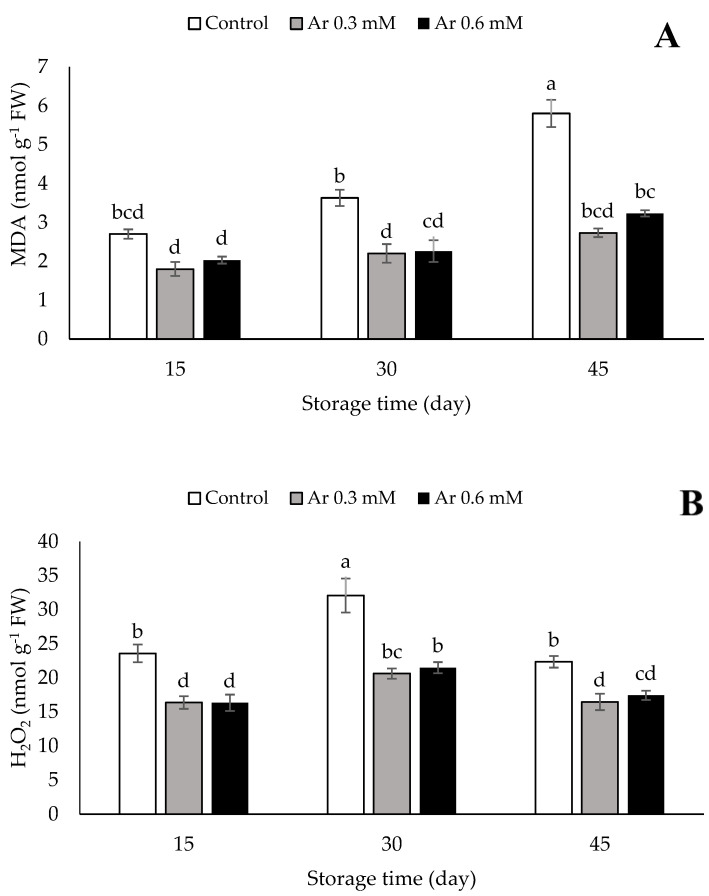
The effect of different levels of postharvest treatment of L-arginine on the MDA (**A**) and H_2_O_2_ (**B**) of persimmon cv. “Karaj” fruits during cold storage (DMRT, *p* ≤ 0.05). The values of data are average of *n* = 3, and bar represents standard error of the means. Different letters above the bars show significant difference at *p* < 0.05 by Duncan’s multiple range test.

The results showed that MDA accumulation was increased in control fruit, but MDA did not increase in treated fruits at 45 d cold storage. No significant differences were observed between L-arginine treatments at 0.3 and 0.6 mM during storage time. These treatments effectively inhibited MDA accumulation in the persimmons at 30 and 45 DAS. The lowest MDA accumulation was 3.23 nmol g^−1^ FW, observed in the treatment of 0.6 mM L-arginine after 45 days of persimmons storage (Figure 2A).

The content of H_2_O_2_ first increased and then reduced during storage time. The increase in H_2_O_2_ was effectively prevented when L-arginine was applied at the rate of 0.3 or 0.6 mM. The lowest rate of H_2_O_2_ synthesis (16.44 nmol g^−1^ FW) was related to the 0.3 mM L-arginine treatment and the highest (22.33 nmol g^−1^ FW) to the control (Figure 2B).

The postharvest accumulation of H_2_O_2_ in fruits increases by chilling stress. H_2_O_2_ is synthesized by the β-peroxidation of fatty acids in glyoxysomes and/or during photorespiration in peroxisomes [30]. Free radicals such as H_2_O_2_ have two functions in plants. They are synthesized at low rates as signal-transporting molecules in the defensive system against stresses [31] and even improve the antioxidant system capacity by increasing the activity of antioxidant enzymes, but they are harmful to plants at high rates so that the antioxidant system scavenges the extra radicals [32]. MDA is the byproduct of the oxidation of unsaturated fatty acids in cell membranes and acts as an indicator of fat peroxidation [33]. The further synthesis of H_2_O_2_ and superoxide results in membrane degradation and the peroxidation of membrane lipids [34]. The L-arginine treatment contributed to membrane conservation by alleviating oxidative stress, thereby inhibiting the increase in MDA and H_2_O_2_ accumulation in white button mushrooms and strawberries at the postharvest period [10,13]. L-arginine treatment significantly prevented the postharvest chilling and browning of potatoes and lettuce [34]. Babalar et al. [8] reported that L-arginine application reduced chilling damage of pomegranate fruits during low-temperature storage by reducing MDA accumulation and electrolyte leakage.

### 3.3. Fruit Tissue Firmness

**Figure 3:**
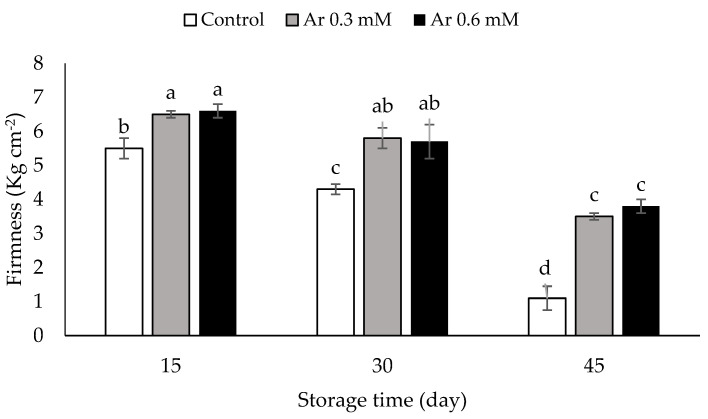
The effect of different levels of postharvest treatment of L-arginine on the firmness of persimmon cv. “Karaj” fruits during cold storage (DMRT, *p* ≤ 0.05). The values of data are average of *n* = 3, and bar represents standard error of the means. Different letters above the bars show significant difference at *p* < 0.05 by Duncan’s multiple range test.

The firmness of fruit tissue was 8.2 kg cm^−2^ at the harvest time, but it decreased in all samples during storage. The L-arginine treatment effectively slowed down the process of the persimmon fruit-softening process during the storage. The treatment of the fruits with 0.3 and 0.6 mM L-arginine effectively contributed to the preservation of the fruit tissue versus the control at 15, 30, and 45 DAS (Figure 3). On 45th day, highest firmness was 3.8 kg cm^−2^, recorded by the treatment of 0.6 mM L-arginine, while the lowest was 1.1 kg cm^−2^ related to the control (Figure 3). Softening and the loss of firmness reduce the quality of persimmons during the postharvest period. The loss of firmness is directly related to cell-wall-decomposing enzymes activities [35]. The activity of pectin methylesterase (PME) and polygalacturonase (PG) enzymes play important roles in reducing fruit tissue during storage [36]. Arginine reduced the expression level of PME and PG, thereby contributing to the preservation of strawberry fruit tissue firmness [37]. Xyloglucan endotransglucosylase/hydrolase (XTH) is an enzyme that prevents the softening of persimmon fruits during storage [38]. The L-arginine treatment increases the expression of the gene responsible for XTH by producing nitric oxide and retards fruit softening [39]. The mushrooms treated with 10 mM L-arginine were firmer than the control during cold storage [13]. Therefore, the persimmon fruits treated with arginine kept their firmness much better than the control fruits.

### 3.4. Weight Loss (%)

**Figure 4:**
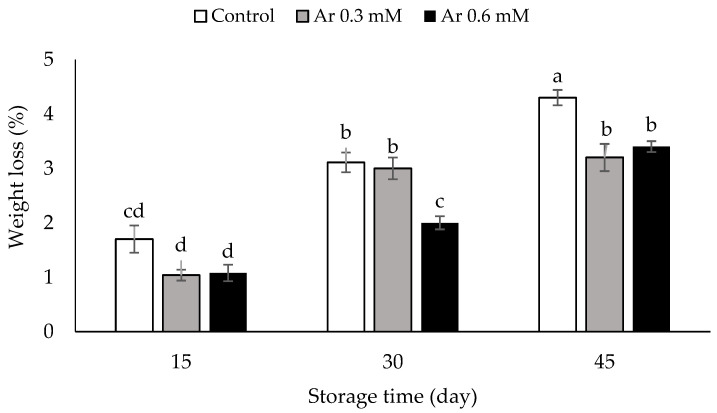
The effect of different levels of postharvest treatment of L-arginine on the weight loss of persimmon cv. “Karaj” fruits during cold storage (DMRT, *p* ≤ 0.05). The values of data are average of *n* = 3, and bar represents standard error of the means. Different letters above the bars show significant difference at *p* < 0.05 by Duncan’s multiple range test.

Weight loss was enhanced in all L-arginine based treatments of persimmons under storage time, but it was greater in fruit of controls than in the treated fruits. The results showed that arginine effectively inhibited the weight loss of persimmon fruits. The samples treated with 0.6 and 0.3 mM L-arginine exhibited significantly lower weight loss (by 3.4 and 3.2%, respectively) than the control at 45 DAS (Figure 4). During the storage time, the weight reduction is because of cell respiration, transpiration, and the metabolic activities of the fruit. L-arginine is a precursor in the biosynthesis of signaling molecules such as nitric oxide [10]. Nitric oxide plays significant role in delaying senescence and improving cellular permeability by eliminating ROS, thereby reducing cell membrane permeability and preventing rapid dehydration of the persimmon fruit and rapid weight loss during storage period [12,13]. Ali et al. [40] showed in “Sandhuri” guava fruit that arginine application decreased the weight loss by the maintenance of membrane integrity during low-temperature storage. The results confirmed the results of the Hasan et al. [12] study on cucumbers treated with L-arginine.

### 3.5. Total Soluble Solids (TSS) and Titratable Acid (TA)

TSS was 16.2% at the harvest time, but it was increased in all samples over the storage time. The treatments had no impact on the prevention of TSS increase. The highest TSS was 20.6%, recorded at 45 DAS, and the lowest was 18.1%, recorded at 15 DAS.

A significant decline was also observed in the TA of the persimmon fruits during storage. TA was 0.59% at the harvest time, but it decreased during storage. The rate of this decline was higher for the control. The lowest TA was noted in control fruits on the 45th day. The treatments did not influence TA, which declined significantly.

TSS and TA are important characteristics that strongly influence the taste, aroma, and flavor of persimmon fruits. TSS is mainly composed of sugars, which increase as fruits mature [41]. TA during storage is used in cell in respiration process and decreases [19]. There are different reports regarding the effect of L-arginine on preventing TSS and TA variations. A research study reported that L-arginine had no impact on the prevention of TSS increase and TA decrease in cucumbers [12]. On the other hand, it has been reported that L-arginine prevented excessive variations in TSS and TA in strawberries and mushrooms by reducing oxidative reactions and retarding senescence [10,13].

### 3.6. Total Soluble Tannins

**Figure 5:**
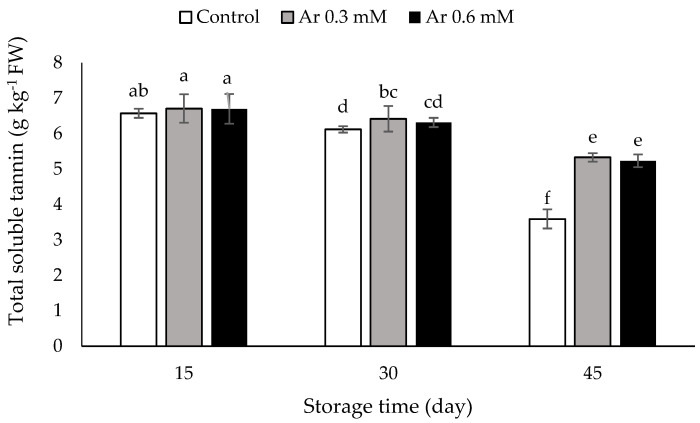
The effect of different levels of postharvest treatment of L-arginine on the total soluble tannins of persimmon cv. “Karaj” fruits during cold storage (DMRT, *p* ≤ 0.05). The values of data are average of *n* = 3, and bar represents standard error of the means. Different letters above the bars show significant difference at *p* < 0.05 by Duncan’s multiple range test.

The total soluble tannins concentration was 5.585 g kg^−1^ FW at the harvest time. The comparison of the means showed that the total soluble tannins were decreased in both the controls and L-arginine applied fruits under storage of cold temperature. No significant differences were observed between 0.3 and 0.6 L-arginine treatment. The highest amount of the soluble tannins was detected in the treatment of 0.3 and 0.6 mM L-arginine at 15, 30, and 45 DAS. The lowest was 3.593 g kg^−1^ FW, detected in the controls, and the highest was 5.330 g kg^−1^ FW and 5.23 g kg^−1^ FW in the treatment of 0.3 and 0.6 mM L-arginine, respectively, on 45th day of storage (Figure 5).

Tannins are classified as phenolic compounds, which are found in many fruits such as persimmon. Moreover, soluble tannins in astringent persimmon fruit are a component of phenol compounds. Soluble tannins in cells are responsible for the astringent taste of persimmon fruits [42]. The fruit of persimmon (cv. “Karaj”) is an astringent type [43]. The amount of soluble tannins is a major characteristic of persimmon quality. At the time of softening and ripening of persimmons, polysaccharide contents and soluble pectins of cell wall are released and joined with tannin contents of soluble nature and altered into an insoluble type, so the astringent taste decreases or is eliminated [44,45]. It has been reported that the L-arginine treatment reduces free radicals and helps cell wall and membrane protection by increasing antioxidant synthesis, thereby preventing the postharvest loss of phenolic compounds [10]. Furthermore, the L-arginine treatment increases stress resistance by synthesizing internal NO and degrading free radicals, which is accompanied by an increase in phenolic compounds [13]. Therefore, L-arginine increases the soluble tannin by increasing the total phenol component.

### 3.7. Total Carotenoids

**Figure 6:**
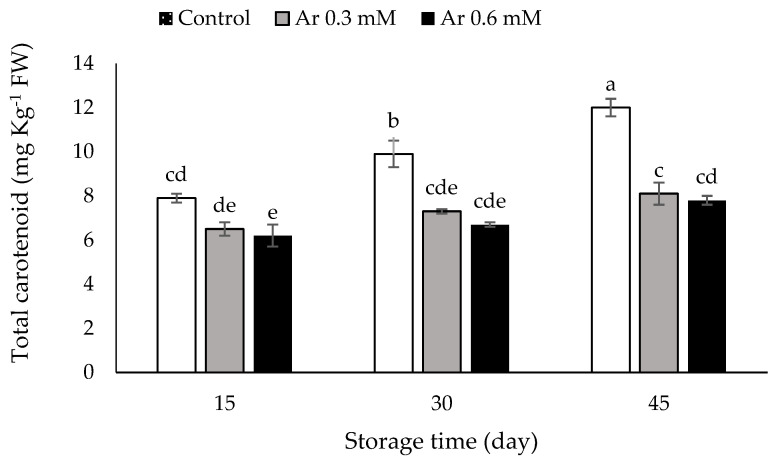
The effect of different levels of postharvest treatment of L-arginine on the total carotenoid of persimmon cv. “Karaj” fruits during cold storage (DMRT, *p* ≤ 0.05). The values of data are average of *n* = 3, and bar represents standard error of the means. Different letters above the bars show significant difference at *p* < 0.05 by Duncan’s multiple range test.

The total carotenoid content, which was 4.5 mg Kg^−1^ FW at the harvest time, increased during the storage time. The treatments effectively prevented the increase in total carotenoid during storage. The L-arginine treatments at a rate of 0.3 and 0.6 mM were effective in preventing the increase in total carotenoids at 15, 30, and 45 DAS. The lowest total carotenoids were 7.8 and 8.1 mg Kg^−1^ FW, recorded at the 0.6 and 0.3 mM L-arginine treatment (Figure 6).

Carotenoids are yellow, orange, and red compounds that have important biological activity in fruits and vegetables and prevent chronic diseases [46]. It has been reported that carotenoids act as secondary pigments in chloroplasts, but their antioxidant role is a more important function [47]. Since persimmon fruits are rich in carotenoid pigments, their fruit color changes from yellow to dark orange during storage. β-cryptoxanthin is an important carotenoid in persimmons that increase during the ripening stage of persimmon fruit [48]. The increase in carotenoid concentration during storage occurs as a consequence of the postharvest ripening process as well as the reduction in TA and firmness. Therefore, the effect of L-arginine treatment on delaying the changes of these parameters could be attributed to a delay of the postharvest ripening process throughout an increment of the activity of the antioxidative enzymes, which are involved in reducing reactive oxygen radicals based damages [12,40].

### 3.8. Catalase (CAT), Superoxide Dismutase (SOD), and Ascorbate Peroxidase (APX)

**Figure 7:**
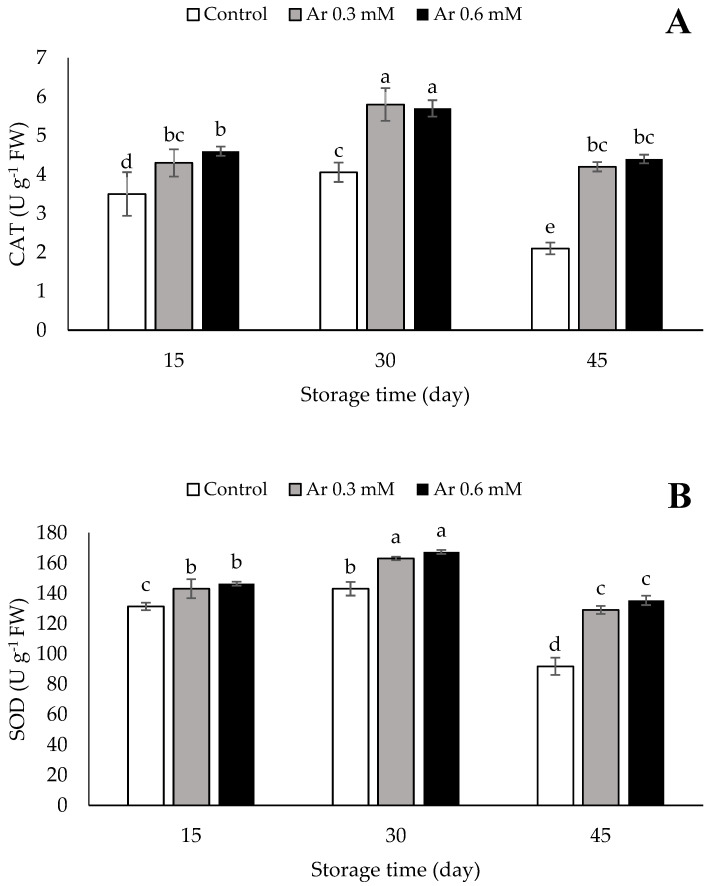

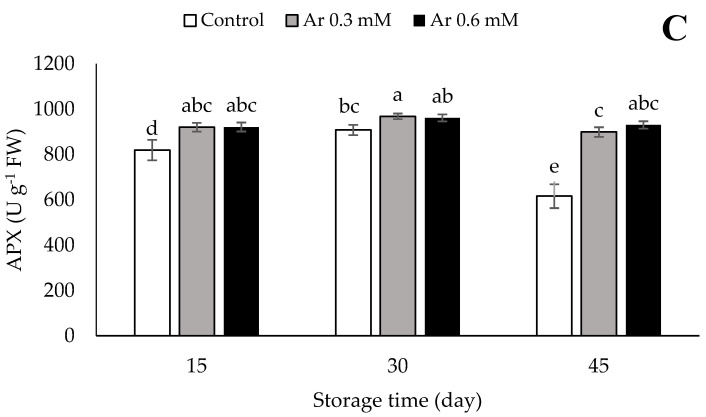
The effect of different levels of postharvest treatment of L-arginine on the CAT (**A**), SOD (**B**), and APX (**C**) of persimmon cv. “Karaj” fruits during cold storage (DMRT, *p* ≤ 0.05). The values of data are average of *n* = 3, and bar represents standard error of the means. Different letters above the bars show significant difference at *p* < 0.05 by Duncan’s multiple range test.

The results showed that the CAT activity first increased and then declined during the storage time. The application of 0.3 and 0.6 mM L-arginine effectively prevented the over-reduction of CAT during storage. No significant variation was noted in the activity of CAT between 0.3 and 0.6 mM L-arginine treatments at 15, 30, and 45 DAS (Figure 7A). According to Figure 7, the SOD activity first increased and then reduced during storage. The L-arginine treatment at the rates of 0.3 and 0.6 mM did not exhibit a significant difference in the SOD activity at 15, 30, and 45 DAS. The L-arginine treatment was effective in hindering the decline of the SOD activity (Figure 7B).

The effect of L-arginine application at 0.3 and 0.6 mM did not differ significantly at 15 and 30 DAS. The highest APX activity (930 and 899 U g^−1^ FW) was observed in the treatments of 0.6 and 0.3 mM L-arginine at the end of storage time. The treatments significantly contributed to preserving the APX activity versus the control during the storage period (Figure 7C).

The antioxidant capacity of fruits and vegetables is correlated to the CAT, SOD, and APX activities as well as non-enzymatic compounds such as phenolic compounds, ascorbic acid, and carotenoids [49]. Stressful conditions and senescence result in the synthesis of free radicals, which are scavenged with antioxidants in fruits. Antioxidant compounds are oxidized by donating electrons to free radicals, thereby decreasing the damage of reactive oxygen species (ROS) [50].

CAT is an important enzyme that inhibits ROS formation. Plants have different mechanisms to cope with ROS. The enzyme CAT is one of the most important mechanisms. CAT is present in the peroxisomes and glyoxysomes of all aerobic cells and protects the cells from the toxic effects of H_2_O_2_ by decomposing it into water and oxygen [51].

The activity of antioxidant enzymes increases during stress, especially the chilling stress [52]. The APX and SOD enzymes have an antioxidative nature with a good potential for removing various ROS. By scavenging free radicals, these enzymes inhibit cell membrane degradation, electrolyte leakage, and peroxidation of membrane lipids [48,53]. SOD is a major O^2−^ scavenger whose enzymatic activity results in the production of H_2_O_2_ and O_2_. Hydrogen peroxide is toxic to cells at high amounts, and thus, some enzymes including APX and CAT regulate H_2_O_2_ content in cells. APX is a specific peroxidase that decomposes H_2_O_2_ through the ascorbate–glutathione cycle. Many studies have indicated that L-arginine treatment stimulates the synthesis of antioxidant enzymes, e.g., CAT, SOD, and APX, during the postharvest period by increasing NO synthesis in fruits [10]. Babalar et al. [8] stated that the L-arginine treatment improved the postharvest activity of CAT, SOD, and APX. The L-arginine treatment effectively stimulated the increase in activities of antioxidative enzyme including SOD in white button mushrooms [13] and tomatoes [54].

### 3.9. Total Antioxidant Capacity

**Figure 8:**
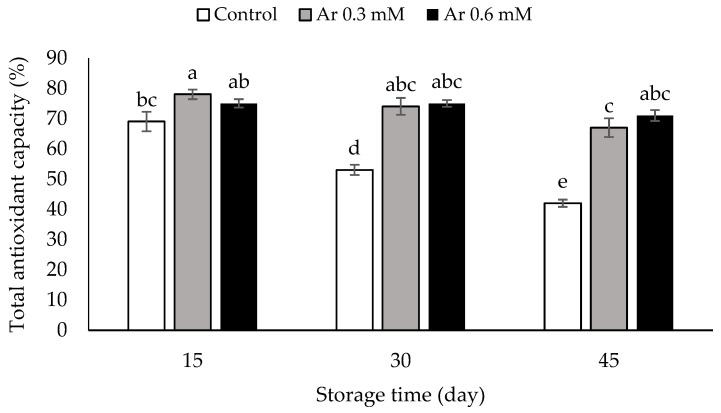
The effect of different levels of postharvest treatment of L-arginine on the total antioxidant capacity of persimmon cv. “Karaj” fruits during cold storage (DMRT, *p* ≤ 0.05). The values of data are average of *n* = 3, and bar represents standard error of the means. Different letters above the bars show significant difference at *p* < 0.05 by Duncan’s multiple range test.

This was decreased during storage in control fruits. The treatments effectively preserved the total antioxidant capacity. Application of 0.3 and 0.6 mM L-arginine did not show a significant difference at 15 and 30 DAS. The highest antioxidant activity was related to the 0.6 and 0.3 mM L-arginine treatment (71%and 67%), and the lowest antioxidant activity was related to the control (42%) at 45th day (Figure 8).

Antioxidants are found in the most fruits that scavenge and neutralize free radicals in the body [55]. Persimmons are rich in antioxidant compounds such as ascorbic acid, carotenoids, and different phenols, including soluble tannins and antioxidant enzymes [56]. Antioxidant compounds play a key role in the postharvest quality of fruits and the scavenging of free radicals during storage [55]. L-arginine treatment significantly contributed to preserving the antioxidant capacity of pomegranates by preserving cell membrane integrity and increasing total phenols and ascorbic acid content [8]. In strawberry fruit, L-arginine was effective in preserving the antioxidant activity during storage at 20 °C [10].

## 4. Conclusions

The application of safe treatments with no side effects for human health, such as L-arginine, helps increase storability and protect the quality of persimmons during cold storage. The treatment of L-arginine at a rate of 0.6 mM was more effective in prolonging persimmon fruits storage life and contributed to decreasing chilling symptoms, preserving firmness, and increasing antioxidant enzyme activities, including CAT, SOD, and APX at 45 DAS. This treatment had no significant effect on TA and TSS. The results showed that most qualitative parameters of the control fruits declined at 45 DAS, whereas the treated fruits could be marketed even after 45 days of storage. Based on the results, it is recommended to commercially apply L-arginine at 0.6 mM to extend the storage life of persimmons.

## Data Availability

Data is contained within the article.
